# Barriers and enablers to collaboration in the mental health system in Sabah, Malaysia: towards a theory of collaboration

**DOI:** 10.1192/bjo.2019.92

**Published:** 2019-12-12

**Authors:** Wendy Shoesmith, Awang Faisal Bin Awang Borhanuddin, Emmanuel Joseph Pereira, Norhayati Nordin, Beena Giridharan, Dawn Forman, Sue Fyfe

**Affiliations:** Associate Professor in Psychiatry, Universiti Malaysia Sabah; and PhD student, Faculty of Business, Curtin University, Malaysia; Research Assistant, Centre for the Promotion of Knowledge and Language Learning, Universiti Malaysia Sabah, Malaysia; Forensic Psychiatrist, Hospital Mesra Bukit Padang, Malaysia; Child and Adolescent Psychiatrist, Hospital Mesra Bukit Padang, Malaysia; Professor of Applied Linguistics and Education and Deputy Pro Vice Chancellor, Curtin University, Malaysia; Professor of Interprofessional Education, Curtin University, Australia; Visiting Professor, University of Derby; Visiting Professor, University of Chichester, UK; and Adjunct Professor at Auckland University of Technology, New Zealand; Epidemiologist, Speech Pathologist and Adjunct Professor, Faculty of Health Sciences, Curtin University, Australia

**Keywords:** Collaboration, global mental health, grounded theory, health care systems, Malaysia

## Abstract

**Background:**

The systems that help people with mental disorders in Malaysia include hospitals, primary care, traditional and religious systems, schools and colleges, employers, families and other community members.

**Aims:**

To better understand collaboration between and within these systems and create a theoretical framework for system development.

**Method:**

A total of 26 focus groups and 27 individual interviews were undertaken with patients, carers, psychiatric hospital staff, primary care and district hospital staff, religious and traditional healers, community leaders, non-governmental organisation workers, and school and college counsellors. Grounded theory methods were used to analyse the data and create a theory of collaboration.

**Results:**

Three themes both defined and enabled collaboration: (a) collaborative behaviours; (b) motivation towards a common goal or value; and (c) autonomy. Three other enablers of collaboration were identified: (d) relatedness (for example trusting, understanding and caring about the other); (e) resources (competence, time, physical resources and opportunities); and (f) motivation for collaboration (weighing up the personal costs versus benefits of acting collaboratively).

**Conclusions:**

The first three themes provided a definition of collaboration in this context: ‘two or more parties working together towards a common goal or value, while maintaining autonomy’. The main barriers to collaboration were lack of autonomy, relatedness, motivation and resources, together with the potential cost of acting collaboratively without reciprocation. Finding ways to change these structural, cultural and organisational features is likely to improve collaboration in this system and improve access to care and outcomes for patients.

Collaborative practice is defined by the World Health Organization as ‘when multiple health workers from different professional backgrounds work together with patients, families, carers and communities to deliver the highest quality of care’.^1^ Collaborative practice includes collaboration between healthcare providers and patients or families, between different professions, between different agencies (for example between healthcare services and social services) and between different parts of the healthcare system (for example between primary care and secondary care). Collaborative practice improves several outcomes in healthcare including: patient and carer satisfaction,^2,3^ functioning,^4^ symptoms,^3–5^ access to care, reduced total costs,^6,7^ length of hospital stay,^6^ hospital admissions,^6^ stress levels among staff^8^ and mortality.^6,8,9^ Although collaboration is a central component of many effective interventions in psychiatry, the concept is inconsistently defined and there is no widely accepted conceptual framework. A literature review of theories of collaboration described five concepts related to collaboration: sharing (for example sharing of resources, shared decision-making), partnership, interdependency, power and processes. Most studies reviewed considered only one type of collaborative relationship (for example interprofessional relationships)^10^ and general theoretical frameworks that incorporate the patient perspective, the interprofessional perspective and the wider community in the collaboration are lacking, especially from non-Western and lower- and middle-income countries.

Sabah is a Malaysian state on the island of Borneo, with a population of approximately 3.8 million. It is socioeconomically and culturally different from the rest of Malaysia, with over 60 different ethnic and linguistic groups, a large Christian population and the highest prevalence of poverty.^11^ Sabah has less health professionals than many other parts of Malaysia, with approximately 0.4 psychiatrists per 100 000 population (compared with 5/100 000 in Kuala Lumpur and a median of 8.2/100 000 in higher-income countries).^12^ Services in the state are largely centralised in a 308-bedded psychiatric hospital and patients in Sabah often seek religious or traditional help before accessing formal healthcare services.^13^ Most staff in the health service have not had formal training in interprofessional collaborative practice and it is not generally part of the working culture or medical and nursing school curricula.^14^ This study was part of a project to create a new model of collaborative practice for the Malaysian psychiatric system. In this first stage we aimed to understand the enablers and barriers to collaboration and to create a conceptual framework to help improve collaboration across the system.

## Method

In this study, grounded theory methodology was used as part of the first phase of a multiphase action research study, so that the theory generated could be used to guide action, using a method similar to that described by Teram *et al*.^15^ The focus in this first phase was to create a theory that could guide the formation of a new model of collaborative care in the system and the interviews and focus groups included exploration of ideas about how the system could be improved as well as what was currently happening in the system. In later stages of the study, a ‘collaborative practice committee’ (which included 11 of the staff who had been originally interviewed, other staff, patients and carers) was formed to act on the research findings and create recommendations to improve collaborative practice in the hospital, which were then reviewed by a nationwide Delphi panel. Some of these recommendations were later implemented in the hospital. These recommendations will be reported on separately.

### Data collection

Data were collected in 2013 and 2014 in Sabah, Malaysia. Semi-structured interviews and focus groups were conducted with healthcare providers (psychiatric services, district hospitals and primary care), patients, carers and community members (religious professionals, traditional healers, non-governmental organisation (NGO) workers, school and college counsellors and village leaders). Participants were interviewed in a focus group where possible, but if this was difficult or inconvenient for the participant then they were interviewed individually. A total of 134 people were interviewed in 27 individual interviews and 26 focus groups of two or more people. Details of the participants are shown in Table 1.

**Table 1:** Details of participants

Participants	*n*
Patients	20
Family members	11
Staff	76
Psychiatric hospital	66
Nurse	23
Medical assistant	13
Specialist	5
Medical officer	6
Occupational therapist	3
Physiotherapist	4
Social worker	1
Counsellor	1
Pharmacist	4
Healthcare assistant	6
Primary care	5
District hospital	5
Community	27
Village leaders	8
School counsellors	5
Religious professionals	7
Traditional healers	3
Non-governmental organisation workers	4
Total	134

A mixture of purposive sampling, theoretical sampling and snowballing was used to recruit participants. Patients and carers were recruited purposively, through recommendation from clinical team members, with consideration given to ensuring that different groups were represented (for example patients with varying length of involvement with services). Posters were also placed in the waiting room, inviting patients to come and give their feedback about the hospital to the research team, but there was no spontaneous response to these. Most patients and carers were interviewed in the psychiatric hospital and were paid a travel allowance, but five were interviewed at home. Staff were mainly selected through purposive sampling, to ensure that each type of professional group was interviewed. Some of the sampling was theoretical, with participants chosen specifically to elaborate emerging categories. Most staff were recruited by the researcher or other staff asking them face to face. In total, 66 staff were interviewed in the psychiatric hospital, with 5 interviewed in district hospitals and 5 interviewed at a primary care conference. Community members were initially selected purposively and then by snowballing, where participants were asked about other people and organisations working with people with mental disorders. Community members were all interviewed in the community.

The decision about the selection of categories of participants was made by team discussion before study commencement and the study stopped when all these categories had been interviewed and core categories were saturated. Decisions about further interviews to explore emerging themes and data saturation was also made by team discussion. No participants refused to participate or dropped out at the point of consent; however, since many participants were recruited through third parties (for example hospital staff referring other hospital staff), it is difficult to calculate a true refusal rate.

Interviews and focus groups lasted between 30 min and 2 h and were conducted in Malay (38 interviews/focus groups) and in English (15 interviews/focus groups). Most interviews and focus groups included only study participants, but five carer interviews took place at home, with community mental health team members present for part of the time. The themes to be explored were developed prior to the interviews/focus groups starting (initial interview questions can be found in supplementary File 1 available at https://doi.org/10.1192/bjo.2019.92). Initial coding was conducted concurrently with data collection and the interview questions altered to explore emerging categories in more detail. Laminated cards with the names of different professional groups (for example ‘occupational therapist’) and other groups that are part of the system (for example ‘patient’, ‘family’, ‘traditional healer’, ‘employer’) were used as prompts for the interviews and focus groups. Participants were asked to arrange the cards to demonstrate the relationship they had with the different groups and were prompted to discuss the relationships as they arranged the cards, including details about the ways that they worked together with each group and their opinion about what was helping or hindering their collaboration with different groups.

### Data analysis

All interviews were audio recorded and transcribed verbatim by the interviewer or research assistant. Brief field notes were also made during the interview. Analysis was conducted in NVivo version 10 using the grounded theory method as described by Urquart.^16^ Interviews were analysed in their original language. During the open-coding phase, the meaning of each phrase was discussed, and a detailed code applied in English. All extracted meanings were coded, with some segments coded multiple times. The detailed codes were regularly inspected and grouped together to form higher-level codes, for example codes ‘bravery in going against hierarchical beliefs’, ‘taking initiative’ and eight other codes were merged together under the code ‘proactivity and assertiveness’, which was eventually merged with other codes to form the code ‘sharing responsibility and accountability’. The constant comparison method was used to compare data within a code and recode or regroup if necessary.^17^ All major themes and subthemes were saturated. The relationship between themes was established by re-examining the data where more than one of the main themes were coded. The COREQ checklist was used to write the research report.^18^ In line with the grounded theory method, the theory was corroborated and expanded by comparing with other theories in the literature (see supplementary File 2 and supplementary Table 1 for more detail).^19^ One major theme was added and another renamed during this literature review stage, with the term ‘relatedness’ derived from self-determination theory.^20^

### Reflexivity and strategies to ensure trustworthiness of interpretations

The majority of interviews were conducted by A.F.B.A.B. (a male linguist and masters student, trained in interview skills by W.S.) and W.S. (a female, UK-trained academic psychiatrist with 7-years post-qualification experience, 6-years clinical work in the hospital and a working relationship with some staff participants). The research interests, assumptions and biases of the principal investigator (W.S.) developed from experience of working in a different system. The assumptions were that a collaborative approach led to better patient care and that collaboration within the system was currently inadequate. Ten of the community interviews were conducted by two research assistants, (social science graduates from the community area and trained in interview skills by W.S.). The participants were told that the purpose of the interview was to better understand what was happening in the mental health system and to find ways of improving collaboration.

The interviews were coded by W.S. and A.F.B.A.B. working together. As the coders were of different cultural and professional backgrounds, greater level of reflexivity resulted as assumptions and biases were more apparent. Memo-ing, journaling and regular team discussions and supervisions were also used to improve reflexivity and some memo examples are provided in supplementary File 3. Repeat interviews were not conducted, but some hospital staff were asked for clarification if important content was difficult to hear on the recordings. Participants were offered the opportunity to read their transcript during the consent process; however, none of them made such a request. Member checking was done by discussion of the findings with the ‘Collaborative Practice Committee’, which was formed to act on the research findings and met between 2016 and 2018.

### Ethical considerations

All participants gave written informed consent. The authors assert that all procedures contributing to this work comply with the ethical standards of the relevant national and institutional committees on human experimentation and with the Helsinki Declaration of 1975, as revised in 2008. All procedures involving human subjects/patients were approved by the Medical Research and Ethics Committee, Ministry of Health Malaysia (NMRR-13-308-14792).

## Results

**Fig. 1:**
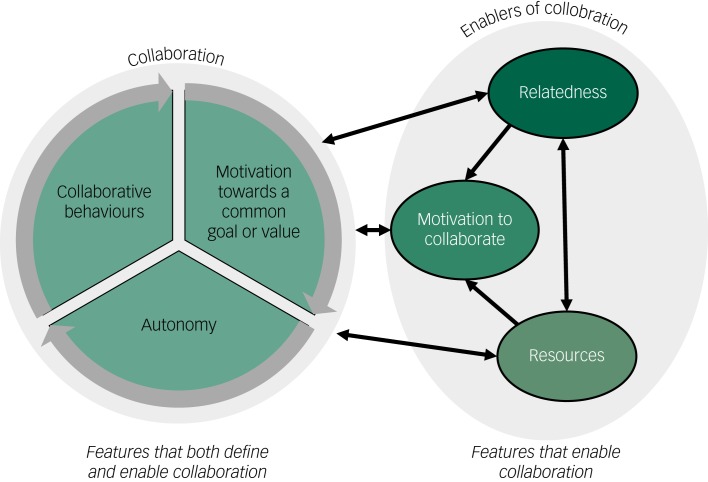
Factors that define and enable collaboration.

There were two core results categories: (a) collaboration and (b) reactions to symptoms. Reactions to symptoms has been published separately.^13^ Data from the various stakeholders coalesced around six themes related to collaboration. There were three features that both defined and enabled collaboration: collaborative behaviours (theme 1); motivation towards a common value or goal (theme 2); and autonomy (theme 3). If any of these features were absent the situation was not collaborative. As the three defining features all mutually reinforced each other, they were also considered to be enablers of collaboration. The other three themes: relatedness (theme 4); resources (theme 5) and motivation to collaborate (theme 6) were considered to be enabling features rather than defining features, as they facilitate collaboration, but they are not required for it (see Fig. 1). The relationships between themes are shown in Appendices 1 and 2.

### Themes that both defined and enabled collaboration

#### Theme 1 (defining and enabling feature): collaborative behaviours

Behaviours defined as collaborative are identified as subthemes shown in Appendix 2. These behaviours included accepting and valuing others' contributions, learning from each other and sharing information, creating and respecting role boundaries and creating goals and a common vision. Sharing was a critical aspect of collaborative behaviour; sharing information, decision-making, responsibility and accountability and sharing experiences, rewards and frustrations were discussed as present or absent in relationships within the system. These behaviours are considered defining and enabling, because when used, further collaboration is generally stimulated. Each collaborative behaviour subtheme was linked with some or all of the five other themes as shown in Appendix 2.

#### Theme 2 (defining and enabling feature): motivation to reach a common goal or value

Motivation to reach a common goal or value was both a defining feature and enabler of collaboration and the lack of this motivation was a barrier to collaboration in this system. Participants mentioned that either they or others were not always motivated to create the best possible outcomes for patients. This was related to the following subthemes (a) general motivation, (b) priorities, (c) goals and values.

##### General motivation

General motivation was related to drive and energy and usually mentioned in relationship to burnout, stress and mental health problems in staff, families or patients. Causes of low motivation described included high workload leading to poor care, lack of resources, lack of autonomy, lack of collaboration from others, lack of progress towards a goal, not working in accordance with values, being asked to do work outside of role, being treated badly by others, lack of support and mental health problems.

##### Priorities

Conflicting priorities included mental health not being prioritised among community members and in primary care. Staff reported that they were expected to implement an ever-increasing number of programmes without extra resources, particularly in primary care.

##### Goals and values

Goals of different professional groups, patients and families were sometimes conflicting and were seldom discussed between them.

‘… just to make patients not being a problem. That's the general objective. We want to treat symptoms but not solving the actual problems…. we are managing them so that they wouldn't be a problem for their family members, they eat well, they don't disturb anyone … I think that's the aim of most families. We are not into making them productive citizens of the country.’

(District Hospital Doctor 1, senior general doctor in a rural area)

Different values were also a cause of frustration and lack of collaboration. Values of autonomy, personal growth and self-actualisation conflicted with values of stability, security and control (such as nursing staff being unable to introduce new activities on the ward because that would reduce monitoring). Values related to caring also conflicted with values related to achievement (of measurable targets).

‘It is so target orientated. You have got 100 targets, 101, and if you miss them, it is like, “why didn't you achieve the target?” It doesn't make sense to me you know. That is why we are losing our holistic patient care.’

(Community Matron 1, senior nurse with supervisory and administrative responsibility working in a rural health centre)

This community matron and several other participants described how not being able to work in a way that was consistent with values (such as caring) led to a loss of motivation in general.

#### Theme 3 (defining and enabling feature): autonomy

Autonomy was considered a defining feature of collaboration because if participants were not acting autonomously, then they were cooperating or complying rather than collaborating (for example patients taking medication because they feel they must). Lack of autonomy, power differences and hierarchy were identified as a major barrier to collaboration by the majority of staff and patients. Almost all participants identified the same power structure, with the doctor at the top, the patient at the bottom and the nursing staff and family somewhere in the middle. They described a centralised, target-driven culture, which gave a feeling of disempowerment to even senior staff and undermined autonomy.

##### Barriers and enablers to autonomy

Appendix 3 summarises the enablers and barriers to autonomy, which consisted of the subthemes behaviours, feelings and thoughts.

The patient below illustrates all these elements.

Interviewer: ‘Have you ever told the doctor that you want to change something, your opinion?’

Patient: ‘Never, maybe next time I will tell them that I want to increase my medication.’

Interviewer: ‘Why did you feel you could not speak out before?’

Patient: ‘I was scared that the doctor would be angry.’

Interviewer: ‘Has the doctor ever been angry?’

Patient: ‘No.’

Interviewer: ‘But you are afraid that it might happen?’

Patient: ‘Yes’.

Interviewer: ‘And you don't feel brave enough?’

Patient: ‘I keep things secret, I lie to the doctor that I always take my medicine. But … it is my fault … I take more medicine sometimes to sleep, because it is hard to sleep.’

(Patient 4, out-patient with one previous admission, treated for 3 years)

The patient is not expressing her opinion (a behaviour) because she is afraid that the doctor will be angry (a feeling). The feelings are likely to be related to a stereotype because the doctor has never been angry with her and she meets a different doctor each visit. Underlying this feeling may be assumed rules: ‘the doctor makes the decisions’, ‘I am expected to follow orders without questioning or complaining’ (beliefs). The doctor and patient are unable to collaborate, and the outcome is that she sometimes runs out of medication and is too worried to ask for more.

##### Consequences of lack of autonomy

Participants from all categories described the hierarchical culture sometimes being detrimental to patient care and a cause of frustration, loss of motivation, poor communication and harsh treatment, which affected staff, patients and carers. The most notable consequence of the lack of autonomy of non-medical staff, patients and carers in the hospital was the way decisions were made. Most staff interviewed described a style of decision-making where the person lower in this hierarchical structure gives information, but not opinions or ideas to the person higher in the hierarchy.

‘It is always the doctor that makes the decision, if he isn't too sure he will talk to a specialist…They will always ask “how is the condition of the patient?”, then go away…they also ask about their progress on the ward, but I have never known them ask “what do you feel should be done?” They don't ask that.’

(Nurse 11, senior nurse working in an acute ward)

This was also seen in the way decisions were made with patients (see the Sharing decision-making and creating a plan section in Appendix 2).

‘Normally they [the doctors] review the patient on the ward, the patient comes in, they ask the patient about how they are today and if they are hearing voices, that they are not seeing anything, when they are finished, the patient leaves, then they write the plan on their own.’

(Nurse 9, junior nurse describing decision-making between doctors and patients)

Several staff described how poor decisions were sometimes made because of this decision-making approach and they wanted system-level culture change.

‘I feel that the culture of not wanting to ask our opinion, I feel it reduces the quality of patient care. They should ask the ward nurse, MA [medical assistant] about their opinion. Because more heads are better than one head. Because I have been working here for 16 years and although there have been changes, the culture is still the same. If this culture continues, there will be no improvement.’

(Medical Assistant 1, senior paramedically trained staff, working in an acute psychiatric ward)

The exception to this was the staff working outside of the hospital (for example in the community mental health team) and allied health staff, who reported that they did give opinions. Doctors reported sometimes wanting opinions from others, but not being given them.

##### Comparisons between different parts of the system

The lack of autonomy that patients faced within the psychiatric system were also present in the family environment. There appeared to be an expectation that families would act in a patriarchal way towards patients. Participants, including patients, implied that one of the most important roles of the family was to ‘control’ or ‘supervise’ the patient.

‘The family must be together to help the patients, the patient has to be led and they need to cooperate to take care of the patient.’

(Village leader 3, rural village leader, with more than 20 years of experience)

Patients are often assumed to be unable to make their own decisions, for example the family can sign the patient into a rehabilitation centre for 2 years, and the patient is not allowed to leave.

‘If the student wanted to leave, and their parents who took them here, so their parents need to agree on this. We cannot let them just go like that. Because their parents send them here so we need to respect the decision of their parents.’

(NGO staff 3: working in the rehabilitation centre, not mental health qualified)

Patients did not describe diminished autonomy in their relationships with religious professionals and nearly all these relationships were described in very positive terms. In contrast, patients frequently described low levels of autonomy in relationships with Bomoh (traditional healers), with patients sometimes describing things being done to them with little explanation.

‘For me it is the religious professionals [who are more effective]. With the Bomoh you just stay quiet, even if they slap you, you just stay quiet.’

(Patient 18, receiving treatment from the hospital for more than 20 years)

### Enablers of collaboration

#### Theme 4 (enabling feature): relatedness

This theme emerged from codes relating to care, caring, supporting, understanding and being connected to others. The subthemes are detailed in Appendix 4. From the descriptions of patients, families and staff, it appeared that relationships between hospital staff and patients and carers were usually friendly, but surface level, where problems were not discussed in-depth.

Interviewer: ‘So is there anyone who knows you, that is close with you or discusses her problems with you?’

Carer: ‘No one. They recognise me from the door, they call my wife, that is all.’

(Carer 7: husband of an in-patient, admitted 2 months earlier)

‘The only thing is that in our set up, because we don't really understand the patient in detail and their needs, like for example we are not really deep enough to understand their problems so not really able to help them in a very structured manner.’

(Specialist 2, working in both out-patient and in-patient settings)

Surface-level relationships, where patients do not discuss problems with staff, sometimes lead to stress and aggression:

‘Stressed patients rarely discuss it with us, but they will show it by becoming aggressive.’

(Medical Assistant 1, senior paramedically trained staff, working in an acute psychiatric ward)

Most patients and carers saw no one in the healthcare system regularly. They described seeing a different doctor every time, both in the psychiatric hospital and in primary care. There was no primary nurse or nurse who knew them well during hospital admissions. Few patients used the names of the staff who had treated them. Carers particularly talked about feeling unsupported, with staff only speaking to them on admission and discharge, but little other contact. The exceptions were when the family were asked permission for electroconvulsive therapy and using clozapine.

The excerpt below, from an interview with junior doctors demonstrates how difficult emotions, associated with relatedness, may become a barrier to relatedness. One of the junior doctors described using numbing as a distancing strategy, which avoids relatedness. The other one describes how relatedness between peers is helping them to manage these feelings.

MO5: ‘There are things that sometimes make me very frustrated, especially when I treat them with tender, loving care with maximum medication, but yet they still relapse, they are still doing the same old, when they come in it is always the same old presentation, so I remember my senior told me that I would feel angry, you will feel frustrated, but eventually you will feel that it is ok.’

MO2: ‘You will feel numb’.

MO5: ‘I haven't feel the numb yet. But I feel from here because we have a lot of support from each other, so if there is any problems we can always, I mean for me, I always voice out, I always tell out, so I listen to a lot of different opinion and I learn at the same time about how to handle such stressful situation, I feel it is quite good lah.’

(Junior doctor interviews (MO5 and MO2): both junior doctors working in in-patient and out-patient departments)

##### Comparisons between different parts of the system

There were parts of the system where relatedness was higher, particularly between doctors and in-patients; between the community mental health team, patients and carers; and between patients and some of the other people who help psychiatric patients.

In contrast with the health system, school and college counsellors described how they often case manage patients and sometimes form close supportive relationships. Some of the religious professionals also described forming close relationships with the people they were treating, with home visits, regular follow-up and intensive involvement at times of crisis. The patients and carers also reported feeling close to religious leaders. One pastor described how he filled in the gaps of the psychiatric system:

‘I mean even when people have already started to see a psychiatrist how often do they see them? But in between, although they take the medication, they still have elements of depression, difficulty coping with emotions and stuff. That is when, even though they are on medication, they still come for counselling with the pastors, just to talk.’

(Pastor 2, pastor from large city church)

#### Theme 5 (enabling feature): resources

Resources were enablers of collaboration and lack of resources were described by many participants as the biggest barrier to collaboration in the healthcare system. We coded competency, time, physical resources, opportunities and collaborative spaces as subthemes. This is summarised in Appendix 5. Lack of resources affected most other features, for example lack of time and training in collaborative skills in staff reduced collaborative behaviours. Lack of the other features further reduced resources, for example lack of collaborative behaviours reduced interprofessional learning.

#### Theme 6 (enabling feature): motivation to collaborate

This theme was related to the process of weighing up whether the potential benefits of collaboration was worth the extra cost and risk that collaboration sometimes entails. It was added after the literature review stage as it was inadequately represented by the other themes and there was adequate data to support the theme. The subthemes were costs of collaboration and benefits of collaboration. Many participants could describe theoretical advantages of collaboration and believed that the whole system would work better if people collaborated better, but also described how the costs of collaborative behaviour (for example lost time) were often greater than the benefits. Some staff described how attempting to collaborate through patient referral with another party had not been effective and this reduced motivation to collaborate again.

‘Sometimes I feel, I refer the patient to the, for example like [name of professional group] I don't much have hope, even though I refer, for the sake of referral, most of the patient will still be the same… so actually not so beneficial.’

(MO2, junior doctor experienced in psychiatry)

For some staff, accountability and risk of blame were some of the main costs of collaborative behaviour, in that any proactivity increases the potential to get into trouble.

‘We already set our mind not to speak… If the ideas are accepted, if there are problems, we will get the backlash. If it is just getting scolded, then never mind, but if it is disciplinary action…’

(Nurse 4, junior nurse, working in out-patient department, previously worked on wards)

## Discussion

### Defining collaboration

Six features enable collaboration in this system: collaborative behaviours, autonomy, motivation towards a common goal or value, relatedness, resources and motivation to collaborate. The first three of these features define collaboration, in that if any of them are absent the situation is not collaborative. From these three themes, we can define collaboration in this system as: two or more parties working together towards a common goal or value, while maintaining autonomy. Working together involves the sharing of information, ideas, opinions, resources, activities, power, rewards, accountability and responsibility, as described in theme 1.

### Autonomy, hierarchy and boundaries

Our definition has differences from other definitions of collaboration, which often do not include autonomy, or stress that collaboration involves interdependence and not autonomy.^10^ Conversely, Wood & Gray,^21^ considered autonomy to be essential to collaboration and that when autonomy is lost it is a merger, not a collaboration. In our study, autonomy was often lacking from one or both parties and these situations cannot be described as collaborative. The defining role of autonomy in collaboration initially appears to be paradoxical, since collaboration also involves interdependence and reduced personal choice. However, autonomy and interdependence do not have to be opposed to each other.^22^ Many definitions of autonomy are based around being able to act in line with internalised values.^23^ If common goals are in line with the values of all parties, they can work together while maintaining autonomy. Some of our participants are not able to work in line with their own internalised values (such as caring), which they experienced as threatening to their autonomy and stressful.

The hierarchical culture in the healthcare setting was a barrier to autonomy and was therefore one of the major barriers to collaboration. Power distance is an indication of hierarchical culture and some studies show it to be particularly high in Malaysia.^24^ High perceived power distance has been shown to reduce incident reporting rates and this is likely to be mediated by reduced psychological safety.^25^ Our study shows that many staff and patients in this system often do not feel psychologically safe enough to speak out if they believe a decision is wrong. Working together, while maintaining autonomy, requires the negotiation and respecting of boundaries and roles.^26^ However, overly rigid boundaries and respect for hierarchy sometimes reduce autonomy in this system and are likely to be preventing optimal outcomes for patients.

### Costs versus benefits of collaboration

Many of our participants wanted change but felt powerless to change the system. For example, in this system both doctors and nurses are aware that collaborating will lead to a better outcome for the patients (a shared goal that both want) and more job satisfaction. However, both parties also believe that the individual costs of attempting to collaborate without reciprocation are too great (described in theme 6). The system is in what is known in game theory as a ‘Nash equilibrium’, whereby neither party is collaborating, since both parties believe that the other party will not collaborate.^27^ Both parties are frustrated, but neither will collaborate while they are in the equilibrium.

### Relatedness

One potential way out of this Nash equilibrium would be to increase relatedness, since repeated interactions allow different equilibria to form as each party is able to develop trust that the other party will act collaboratively.^28^ The role of relatedness in psychiatry is well established, in that the therapeutic alliance with a healthcare professional is one of the best predictors of outcome in psychiatric illness.^29^ The organisational literature puts a high value on trusting relationships in collaboration and recognise that these take time to develop.^30^ The staff interviewed rarely discussed topics related to the therapeutic alliance and there appears to be a lack of awareness of its importance (see Appendix 4, barriers to relatedness). Most staff in the system have not received specific training in mental health and had trained in settings that are geared towards the treatment of short-term episodes of physical illness, where relationships are less critical to the outcome. It also appears that some staff are unsure about how to negotiate and maintain boundaries, while having a genuine, caring relationship with a patient. Reorganising systems to improve continuity of care and training staff in these skills is likely to improve outcomes.

### Resources

One of most frequently mentioned barriers was a lack of resources, which meant that participants did not have the time to collaborate. The system is under pressure, with high numbers of patients for the number of staff. This reduces the ability of the people in the system to improve anything, causing a powerlessness that leads to a loss of motivation. Change may improve efficiency in the long term, but normally needs increased resources in the short term.^30^ This short-term loss of efficiency is much more difficult to tolerate in an overloaded system. While more resources are available outside of the hospital, for example school counsellors, these people are rarely involved in planning patient care. The lack of time for collaboration means that those resources outside the hospital system are not fully utilised.

### Implications

Low levels of autonomy, relatedness and resources are the main barriers to genuine collaborative relationships in this system. The low level of collaboration in the system appears to be causing poor outcomes for some patients and their families, reducing job satisfaction in staff and underutilising the value of the relationships with religious and community leaders and school counsellors. The features that influence collaboration are all intimately connected to one another and any successful solution will probably need to act on all these features at the same time. Changing one of these features alone may not achieve significant change, but training of staff in collaborative skills and reorganising systems to improve continuity of care would be useful first steps towards improving collaboration in the system. Many staff are aware of the problems and have some ideas about how to fix them but feel powerless to change anything. The inertia in the system means that paradoxically culture change may need to be imposed through a top-down approach, to create conditions where autonomy and relatedness are possible and collaborative behaviours are supported and encouraged.

### Strengths and limitations

The strengths of this study were the number and wide range of people interviewed and that collaboration was considered from multiple perspectives. The use of cards with different roles during the interview allowed participants to focus on relationships and allowed conversations to progress to a greater depth and level of candour. Limitations included that the principal investigator worked in the hospital and had a relationship with many of the participants in this study. This may have created biases and limited what some of the participants were willing to talk about, but also meant that the research team had a more in-depth understanding of the system.

## Data Availability

W.S. and A.F.B.A.B. had full access to all data. W.S. currently has the data and has ongoing access. Other authors had access to the coding tree and important non-identifiable data. Selected, non-identifiable data are available from the authors on request.

## Supplementary material

For supplementary material accompanying this paper visit http://dx.doi.org/10.1192/bjo.2019.92. click here to view supplementary material

## Appendix

**Appendix 1:** Relationship between themes (also see Appendix 2 that shows relationships with collaborative behaviours)

Relationship between…	Examples
Relatedness and resources	Staff not knowing patients well reduces their competence to work with a particular patient (for example they are unaware of their educational needs). Time used inefficiently, since the same information is collected from the patient repetitively. Low resources means staff do not have time to gain an in-depth understanding of patients. Lack of mental health training leads to difficulties managing boundaries and the difficult emotions associated with relationships, leading to distancing strategies.
Relatedness and motivation towards goal/value	Caring about a patient increases motivation towards common goals and values. Relatedness reduces burnout in staff and increases motivation. Working towards goals/values together increases relatedness.
Relatedness and autonomy	Relatedness increases autonomy in both parties, by reducing hierarchy reinforcing stereotypes. Autonomy of staff allows them to work in line with relatedness-based values (for example caring).
Resources and autonomy	Lack of time and resources reduce the autonomy of staff to be able to act in line with their values. If staff are able to make decisions autonomously, they can be more efficient.
Resources and motivation	Low resources (time, training, physical resources) means people get frustrated and give up. Staff are unable to work towards goals if resources are too low for goals to be reached. Low motivation means resources do not improve – staff, patients and carers are not engaged and stop learning and building.
Relatedness and motivation to collaborate	Staff that do not trust each other do not believe the other person will reciprocate if they attempt to collaborate.
Resources and motivation to collaborate	Attempting to collaborate risks losing resources (for example time), without getting any closer to goals. Low resources reduce the risk-taking in attempting to collaborate.

**Appendix 2:** Collaborative behaviours and relationship with other themes (theme 1)

Collaborative behaviour	Examples and relationship with other themes
Accepting and valuing the contribution of the other	Asking for help, referring to each other, valuing and appreciating each other. Hospital staff referred to each other. Staff sometimes mentioned feeling devalued or not listened to when attempting to make contributions. Relationship with: autonomy↔, relatedness↔, resources↔, motivation to collaborate↔
Creating goals and a common vision	Described theoretically by some participants as being important for collaboration. Participants did not describe a regular process by which this happens, and some reported that it does not happen in the hospital. ‘I think what we lack is that we sometimes don't see a common vision for our patient, and I think also each person understands mental illness in a different way, so that is where the main obstacle comes…Because we come from different backgrounds. How to unite these people of different backgrounds will be one major challenge.’ (Specialist 3) Relationship with: motivation towards common goal/value↔, relatedness↔
Creating and respecting boundaries and roles	Inside the hospital strong role boundaries were described, which were sometimes rigid – for example the role of doctor as ‘decision-maker’ (see Theme 3). Some participants, particularly school councillors reported that their role was not understood or respected. Some crossing of role boundaries by doctors, was causing frustration. ‘We did an assessment…the patient didn't have a problem that needed chest physio…So when I discussed with the doctor he was harsh and said to just do it. Although the patient from the assessment really didn't need it.’ (Physiotherapist 1) Relationship with: autonomy↔+–, relatedness↔+–, resources↔+–, motivation to collaborate↔+– (‘Respecting boundaries’ was one of the ways that autonomy was maintained, but sometimes in conflict with other collaborative behaviours, for example maintaining boundaries sometimes reduced sharing of information, resources and responsibility)
Sharing information and learning from each other	Communication was frequently one way, with just a brief referral form. Information was sometimes not shared, for example primary care staff not being aware that a patient had been admitted or discharged. From primary care interview: Family medicine specialist 1: ‘The department sends back a small slip saying, “thank-you for your referral we are currently seeing and following up this patient”.’ Student health centre doctor: ‘If you are very lucky, you will get that.’ Family medicine specialist 2: ‘If you are very fortunate.’ Student health centre doctor: ‘Most of the time nothing.’ Family medicine specialist 1: ‘Normally no diagnosis.’ Staff discussed training community members and psychoeducation of patients and families. Traditional healers wanted hospital staff to learn about them. Some staff discussed learning from each other, mainly from staff of the same profession. Relationship with: autonomy↔, relatedness↔, resources↔
Sharing decision-making and creating a plan	Included eliciting opinions, sharing opinions, listening and coming to a decision together. Shared decision-making was described outside the hospital (for example in families making decisions about seeking help for the first time) between members of the same profession (normally between specialists) and in the community mental health team, but decision-making inside the hospital was rarely shared (see theme 3). Relationship with: motivation to common goal/value↔, autonomy↔, relatedness↔, motivation to collaborate↔, resources←,→+–
Sharing responsibility and accountability	Included proactivity and assertiveness, autonomous helping and following the agreed plan. Staff in the same profession helped each other if one of their colleagues needed help. Some staff deliberately withheld ideas and were not proactive, to avoid blame (see Theme 6). Relationship with: autonomy↔+–, relatedness↔+–, motivation to collaborate↔+–
Sharing experiences, rewards and frustrations	Sharing feelings of enjoyment, stress or frustration. From junior doctor interview: ‘Take for example, some of the staff nurse, they do offer some consolation, “it is ok, this patient is always like this”.’ (MO1) Relationship with: motivation towards common goal/value→, relatedness↔, motivation to collaborate→
Sharing activities and resources	Community participants described joint events, between the hospital and non-governmental organisation workers, religious leaders and other community leaders. Community mental health staff described joint home visits. Relationship with: resources↔, relatedness↔, motivation to collaborate↔

→, collaborative behaviour increases the feature; ←, feature increases the collaborative behaviour; **+–**, relationship is both positive and negative (further information on relationships in supplementary File 3).

**Appendix 3:** Enablers and barriers to autonomy (theme 3)

	Enablers	Barriers
Beliefs	Assumed rules (for example ‘Patients should be given choices’). Beliefs about competence (for example ‘Educated patients are able to understand’). Stereotyping (for example ‘Nurses are friendly’). Beliefs about independence and personal responsibility.	Assumed rules (for example ‘The doctor should make the decisions’, ‘The family should supervise and control the patient’). Beliefs about lack of competence (for example ‘The doctor knows more than me’). Stereotyping (for example ‘Doctors are fierce’). Beliefs about dependency.
Feelings	Confidence, bravery, respect, feeling respected, acceptance, feeling accepted, feeling responsible, feeling recognised, feeling connected.	Fear, feeling looked down on, fear of punishment or social disapproval if not following assumed rules. Descriptions of pride and anger in the person higher in the hierarchy (by the person lower in the hierarchy).
Behaviours	Collaborative behaviours: shared decision-making; respecting, accepting and validating the contribution of the other; respecting boundaries and roles; sharing information; sharing responsibility and accountability.	People perceived to be higher in the hierarchy: monitoring, restricting, contingencies (punishments or rewards), giving directives, leaving out of decision-making and non-collaborative communication (for example not listening). People perceived to be lower in the hierarchy: ingratiating, not expressing opinions or ideas, not making requests and not setting boundaries with people higher in the hierarchy. Physical cues, symbols and non-verbal communication (for example the number of doctors in the ward round intimidating the patient).

**Appendix 4:** Benefits, enablers and barriers to relatedness (theme 4)

Subtheme	Examples
Components of relatedness	Caring, support, trust, depth of relationship and acceptance.
Benefits of relatedness	Understanding each other, understanding roles, understanding the situation and problems to be solved, increased autonomy in the relationship, feeling supported, open communication, increased influence, enjoyment of the relationship, taking responsibility and better outcomes. ‘For example, the nurses are my friends and that makes it easier to discuss patients. Not like gossip, but for the benefit of the patients.’ (Occupational Therapist 1)
Enablers of relatedness	Having a relationship with the same person (for example seeing the same doctor on each visit), collaborative behaviours, competency in relatedness, feeling supported, proactivity (for example home visits, calling a patient who does not come for an appointment), regularity and frequency of meetings and accessibility (for example being able to contact when needed)
Barriers to relatedness	Resources (described in theme 5). Avoidance of difficult emotions associated with relationships, for example patients described how shame about the illness, guilt about being a burden and fears about rejection lead to deliberate distancing from others. Carers described disappointment, sadness, frustration, guilt, shame and anger associated with the relationship with the patient. Staff described sometimes how being unable to provide adequate care for a patient that they cared about (normally because of lack of resources) leads to feelings of shame in the staff involved. Lack of support. For example staff reported feeling blamed by people higher up in the hierarchy, rather than being supported by them to form closer relationships with patients. Fears about managing boundaries. For example staff reported a fear of families or patients become dependent or ‘spoiled’ if they showed too much care. Relatedness not being valued. For example staff described a task-oriented system, where relationships mattered less than tasks, routines and targets. Few staff discussed the benefits of a therapeutic alliance with patients.

**Appendix 5:** The effect of resources on collaboration (theme 5)

Subtheme	Examples
Time	Healthcare staff report not enough time for collaboration. Sometimes related to autonomy in staff (time spent meeting and documenting targets meant less time to spend with patients). ‘We don't spend more than 5 min. It's always less than 5 min…We do not give them the room or the time and opportunity for them to describe their topic. So usually we don't spend a lot of time. I think this is 2 main questions that we ask, “do you sleep well?” or “do you have a good appetite?” then that's it, finish. It's more like fire fighting.’ (District hospital doctor 1)
Competencies	Mental health competencies – perceived lack of knowledge in one party reduced collaborative behaviour (for example patients not getting involved with decisions about their care, because they believed the doctor knows more). Specialised competencies – the competencies of a particular profession (for example staff reported that collaborative practice was difficult because of a lack of psychologists). Situation specific competencies – knowledge about a particular patient or a particular community. Lack of relatedness reduced this type of competency. Collaborative competencies – for example staff described lack of skills in collaborative behaviours in others.
Physical resources and opportunities	Resources and opportunities needed to meet goals. Inadequate resources leads to loss of motivation, which reduces collaboration. For example lack of work opportunities for patients causes loss of motivation in staff and patients and reduces collaboration to reach the goal of returning to work.
Collaborative spaces	Current collaborative spaces: meetings, ward rounds. Suggested ways to improve collaboration: computer system, patient handheld records, organisation into teams. Current collaborative spaces only experienced as collaborative by higher-level staff. Lower-level staff and patients did not attend or did not experience as collaborative.
