# Supplementary material for: Barriers and enablers to collaboration in the mental health system in Sabah, Malaysia: towards a theory of collaboration
Source: BJPsych Open. 2019 Dec 12;6(1):e4. doi: 10.1192/bjo.2019.92 (PMC7001484; doi:10.1192/bjo.2019.92)
Supplement: Supplementary file 1 [file S2056472419000929sup001.docx]

# Supplementary File 1

# Interview schedule example – staff focus group

*(These questions evolved with later interviews, as concepts that needed exploring became clearer).*

1. What do you understand about the concept of collaboration?

Give definition of collaboration, if not sure what it means.

1. Can you tell us about the collaboration here in this hospital?

- If mention eg ward round- What normally happens in the ward round?

To start the discussion, we would like you to take part in an exercise which to identify your relationship with the parties. (Show cards with the names of different professions and groups. Cover table with large piece of paper). Put yourself in the middle (card with interviewees’ group/ profession). Can you arrange the cards to show your relationships with other groups? Can you draw lines to show the relationship between you and the other parties?

1. Talk us through these different relationships and what made you arrange it in this way (if not already discussed during the arranging)

Follow up questions (skip if already covered)

1. We will start with you in the middle.
   - Can you tell us about your job and the scope of your job and how you help patients?
   - Can you tell me some things that you would like to be able to do but you can't in order to help the patients?
   - What are the barriers that prevent you from doing it?
2. We will now discuss this relationship (pointing at line between cards, going through each in turn):
   - Can you tell me more about your relationship with this group
   - Typically how often you talk / talk about a patient?
   - What are the barriers of working with them?
3. I now want to ask you more about your group’s relationship with the patients. Can you describe this?
   Follow up if necessary

- Can you describe communication between your group and the patients?
- Do you feel that you develop a therapeutic relationship with a patient (explore importance of this to the participant)?
- Does each patient have a specific nurse / MA to work with?
- Do you believe it would help the patients if they did?
- How do you help a patient if they become distressed

1. I want to ask some more questions about treatment planning and decision making. How is the treatment plan made? How are decisions made about patient care?
   - Out of the people here, who normally makes the treatment plan? Who else is involved?
   - Do you exchange ideas and opinions or only information?
   - Who has the power to decide?

- What happens normally happens when the patient becomes angry or agitated

1. If not covered:
   - What groups are the easiest to communicate with?
   - What group are the hardest to communicate with?
2. In addition to those you have indicated, do you have any relationship at all with the groups that you have not indicated? What is the barrier to working with them?
3. What is important element for a collaborative practice to succeed and the barriers to it?
4. In your opinion what are the things that can prevent successful collaboration? What helps or would help improve collaboration here?
5. What do you think about the treatment provided here in the hospital?
6. What are the changes that need to be done to provide better treatment?

Supplementary File 2

Methods of Literature Review to Expand the Theory
A literature search was conducted using “barriers to collaboration”, “collaboration theory” as search terms, and papers were purposively sampled from a variety of fields (see supplementary Table 1). The papers were then coded in a separate NVivo project, using our themes that emerged from the original data as a template. Further theoretical sampling of papers was done until higher level themes were also saturated in this project. This exercise did not significantly change the theory, but it did lead to one further main theme and a renaming of some of the codes. The original data were then re-examined to consider whether our data supported the new theme. A further round of coding was done to accommodate the new theme. Further collection of data was not necessary since the new theme was already saturated with the existing data.

Supplementary Table 1 - List of Literature Coded

| Mental health | Beecher, 2009; Kaas, Lee, & Peitzman, 2003 |
| --- | --- |
| Healthcare | Aein et al., 2011; Bosque, 2011; Sørensen et al., 2013 |
| Interagency working | Downie et al., 2001; Gajda, 2004; Price-Robertson, 2012 |
| Tourism | Arnaboldi & Spiller, 2011; Jamal & Stronza, 2009 |
| Engineering and design | Arsenyan et al., 2011; Daoudi & Bourgault, 2012; Guo & Feng, 2010; Spinuzzi, 2014 |
| Research collaboration | Bennett & Gadlin, 2012; Harley & Blismas, 2010 |
| Child protection | Appleton et al., 2013 |
| Education | Muijs et al., 2011; Ryan and Deci, 2000 |
| Writing | Duffy, 2014, |
| Management and organization | Gajda, 2004; Huxham & Vangen, 2004; Vangen, 2016 |
| Music | Mazzola & Cherlin, 2009; Sawyer, 2006 |

# Supplementary File 3

# Examples of memos describing relationships between themes

**Name:** Relationship between autonomy and relatedness

Autonomy to relatedness

Hospital procedures meant relationships were not being formed.

Carer found ward staff to be unsupportive because they would not give him any information, they just always told him to ask the doctor. The lack of autonomy of the ward staff was reducing their support of the carer.

Ward staff do not contact family members unless instructed by a doctor.

Healthcare assistants feeling blamed (e.g. when a patient hits them) also makes them feel unsupported. This strengthens the belief that patients have more rights than them and are more cared about than them.

Some staff appeared to want relatedness but beliefs about professionalism and not having control over own time was stopping them.

Nurses describe working with a team member that is troublesome. They don't have control over who they work with and this is causing problems in relatedness and motivation.

Patients describe feeling close to nursing staff at the same time as describing control by them on the ward. One patient described feeling close to ward nurses because he relied on them for everything on the ward.

The cycle of control leads to ingratiating behaviour in the person lower in the hierarchy, which leads to feelings that they are close. Ingratiating behaviour involves putting their needs and values below the other persons - e.g. belief that the district officer is unable to deal with the problems of the homeless mentally ill because they are ingratiating the politicians.

Catholic sister describes how she proactively goes into the villages to find people that need her help, she is working autonomously and forming relationships.

One patient describes how he chooses (autonomy) to distance himself from others (relatedness), so that people don't hate him, because he is worried that he is ill and might say the wrong thing. However, he also reports that this causes him to feel unaccepted and unliked.

Relatedness to autonomy

People that know each other less likely to use stereotypes - part of the cycle of control vs autonomy.

People that know each other’s strengths and weaknesses were more likely to see the other as competent.

Pharmacist describes how not having relatedness on the ward means that they can't find the person responsible if there is a medication error. i.e. lack of relatedness mitigates against the blame culture.

Senior staff describe how forming relationships with patients and families and educating them reduces fear (eg of ECT, entering the ward) which makes them more open (collaborative behaviour).

Specialist describes how weekly meetings with the same staff (relatedness), could potentially develop their confidence (resources and autonomy) and mean that they could have meaningful discussions about a patient that they all know well. Specialist reports that the lack of relatedness between nurses and patients means that the nurses are unable to give opinions.

Nurses describe how patients are brave enough to express opinions with staff that they know well (MA ward interview).

Nurses describe how fear of the patients is exacerbated by being blamed by people in authority. If something goes wrong, they will be blamed, so that makes them distance themselves from the patients.

Bridges reduce the effect of hierarchy. E.g. CMHT nurses develop a relationship with the patient and are then able to discuss with the doctor what the patient is afraid to discuss themselves. This is not happening on the wards where the patient is already closer to the doctor and patients do not have relationships with specific nurses. However, bridges can also reduce collaborative behaviour and relatedness between the people at either end of the bridge. When messages go through someone else, they become one-way and the flow of feedback can be reduced - e.g. when patients tell pharmacists or ward nurses that they want to change medication and the pharmacist or ward nurse talk to the doctor then persuade them that the doctors choice is correct. The bridge gets in the way of the two people at each end of the bridge.

People are afraid of people becoming dependent - i.e. reducing autonomy. Relatedness without boundaries leads to autonomy reducing.

Carer was able to set boundaries (increase her own autonomy) through the support of the CMHT staff that were well known to her. She was particularly close to a doctor who had a relative with a mental disorder (self-disclosure).

**Linked Item**

Relationships\\Autonomy (Two way relationship) Relatedness

**Name:** Relationship between autonomy and collaborative behaviour

Autonomy to collaborative behaviour

Autonomy is part of collaborative behaviour.

People are not collaborating unless both are autonomous, they are cooperating.

Sometimes neither party had autonomy, in which case a decision is made by someone else - e.g. nurses complaining that the MOs can't make any decisions and everything has to be decided by the specialist.

Healthcare assistants describe how if they give an explanation of an incident the people in authority accuse them of making excuses (blaming- hierarchical behaviour). Their viewpoint and contribution are not valued (non- collaborative behaviour). The people in authority are not sharing responsibility (non-collaborative), since they do not want to look at factors beyond the individual level. They feel the people in authority do not know what is happening on the ground.

In a hierarchical relationship then giving opinions (a collaborative behaviour) is seen by some as arguing (breaking an assumed rule).

Doctors giving staff orders (hierarchical behaviour) to carry out tasks which do not utilise their training (e.g. chasing results from the lab, which can take many hours) makes them feel devalued.

The patient says that they preferred a previous medication to the pharmacist. The pharmacist tells the doctor. The doctor tells the pharmacist the new one is better. The pharmacist explains this back to the patient.

The number of people in the ward round intimidates the patient and makes them not honest (a non- collaborative behaviour).

The pharmacist calls the doctors to say they have written the prescription wrongly. The doctor is sometimes angry, but they do it anyway.

The family say that they want to try the Bomoh (expressing opinion), so the hospital gives home trial leave for the family to do that (respecting their decision, not restricting).

Doctors making the decisions means that the OT is sometimes a passive receiver of referrals, without discussion.

Medical officers say they have no feedback from other staff

Lack of autonomy in nurses reduces spontaneous helping of patients (a collaborative behaviour)

Collaborative behaviour to autonomy

Collaborative behaviour slows down the cycle of control versus hierarchy. Listening, assertiveness etc reduce fear and change beliefs, e.g. the belief that the doctor will be angry if anyone else takes part in decision making

The physio giving the forensic patients choices reduces the sense of hierarchy

The physio, OT and social worker give their opinion to the doctor. The doctor accepts their opinion, and this reduces the sense of hierarchy.

When patients express their opinion, it reduces the sense of hierarchy

When people act in a way which is 'routine' i.e., they do what they always do without thinking (i.e. little autonomy), this enables the hierarchy

**Linked Item**

Relationships\\Autonomy (Two way relationship) Collaborative behaviour

**Name:** Relationship of resources to relatedness

Resources to relatedness

Staff don't have time to spend with the patient, so can't make relationships. Pastor + student health centre doctor describes how they fill the gap.

Patients are not seen frequently enough for relatedness- the counsellor and the pastor describe how they fill the gap. They also imply that patients do not feel they can contact if they have problems.

Increasing relatedness may reduce flexibility so waiting times may increase (from collaborative practice committee)

Not enough resources to see a counsellor

When community mental health team came along in district hospital, able to do defaulter tracing, a relatedness behaviour.

The nurse describes how some families are disappointed in the ward and want better for their relative, e.g. a single room. The nurse helps the doctor to manage the family.

It takes time to build a therapeutic alliance.

Lack of community resources (patient can't get a job) leads to family not valuing them (valuing contribution) which leads to reduction of support (relatedness).

Relatedness to resources

Will reduce doctors having to take a history several times.

Will make more currently untapped community resources available - e.g. school counsellors.

Will reduce burnout and engagement.

Stop patients from falling through the net.

When families give up, then more resources are needed, because the family will want the patient to be in hospital.

Ward carer feels unsupported because the ward staff are not aware of her educational needs, so she feels unprepared for her husband's discharge.

Understanding a patient by caring for them (relatedness) gives you competence in communicating with them.

Ward MAs talk about families who care about their family member wanting extra resources- wanting to ‘treat them like a baby’.

Lack of competency/resources + relatedness = shame.

**Linked Item**

Relationships\\Resources (Two way relationship) Relatedness

**Name:** Relationship between resources and motivation

Resources to motivation

No resources - people get frustrated and give up. Includes lack of competency in self or others. If patients don't get better- same result. No results leads to loss of motivation.

Feeling of shame if unable to provide appropriate services due to lack of competency (community matron) or lack of resources.

Motivation to resources

Loss of motivation causes resources to reduce - staff, patients, carers not engaged. People stop learning and building.

**Linked Item**

Relationships\\Motivation towards common goal or value (Two way relationship) Resources

**Name:** Relationship between resources and collaborative behaviours

Resources to collaborative behaviours

People need collaborative spaces and collaborative competencies to collaborate.

If no resources available then no motivation to reach goal, so no collaboration.

If people have mental health knowledge, then they will refer (collaborative behaviour) (primary care interview).

Doctors listen to attendants (a collaborative behaviour) because they appreciate the attendant’s knowledge about the patient.

The only collaborative space is the ward round, which has too many people (pharmacist)

Specialist can't value the special skills of the counsellor or social work, because they are doing general tasks (e.g. making identity cards) rather than being able to use their special skills. The resources are too low for people to use their skills.

Patient isn't meeting doctor often enough, so counsellor will find doctor to say the medication doesn't suit (acting as a bridge - advocacy).

Medical assistants help the doctors against 'high class' families that want more than the resources will allow - ?protecting them from shame and guilt.

From community mental health team – the patient knows more about their medication, because they are the one's eating it - they are respecting knowledge (competence to collaborative behaviour) and listening to the patient (collaborative behaviour)

Collaborative behaviours to resources

Increasing resources (including own competency) is a goal of collaboration

Collaboration leads to better resources.

People learn from one another.

Traditional healers want doctors to learn about traditional medicine (a collaborative behaviour) so that they can increase their knowledge about the other (resources) and then collaborate better. The traditional healers believe that lots of problems are happening because healthcare staff do not understand spiritual matters.

**Linked Item**

Relationships\\Resources (Two way relationship) Collaborative behaviours

**Name:** Relationship between motivation and collaborative behaviours

Motivation to collaboration

Working together to reach a common goal is the definition of collaboration that several participants used. Some described common values, or not having common values reducing collaboration.

Collaboration to motivation

Non collaborative behaviour was demotivating - e.g. not following agreed plan by other staff, patients, carers. (staff not bringing patients to physio)

When one person shows that the skills of the other are not valued, it is demotivating - e.g. college counsellors asked to do multiple admin tasks, because the college does not understand the role of the counsellor. Ward nurses being asked to chase results, rather than care for patients. Allied health staff and nurses being told how to do their job by doctors

People enjoyed collaborative relationships.

Collaborative environments e.g. CMHT was where staff appeared to be the most motivated.

**Linked Item**

Relationships\\Motivation towards common goal or value (Two way relationship) Collaborative behaviours

**Name:** Relationship between motivation and relatedness

Motivation to relatedness

Relatedness has values associated with it (love, caring, compassion).

People who are motivated by these values will seek relatedness in their work.

One doctor describes numbing - cutting themselves off. Avoiding relatedness is a response to stress.

One of the reasons that nurses don't want to be accountable (which appears unmotivated) is to protect the relatedness with patients - it is the doctor that is ordering this painful injection, not me.

Relatedness to motivation:

When staff are unable to act in accordance with their relatedness values they became frustrated and demotivated. Healthcare staff that appear to value caring get stressed when they can't provide good quality care. When staff develop a relationship with a patient and are not able to provide for them, they exposed to shame and guilt - eg community matrons. Staff may be avoiding relatedness in order to avoid shame and guilt.

Staff working in shifts and no-one being responsible for the patient means that staff blame the previous shift if there is a medication error. Lack of relatedness leads to lack of accountability (pharmacist interview)

Staff describe frustration when family do not visit, since it goes against their values of caring (MA ward transcript)

When staff are supported they are better motivated.

When staff don't know each other well enough they don't have a common vision, so they are not motivated to the same goal or values (Specialist 3)

Patients and doctors get demotivated when the patient puts in the effort and the family does not support (MO interview) also related to resources- if family doesn't support the resources are not there.

Ward staff can't support doctors because they are not looking after fixed patients (MO interview).

Families that do not feel supported will become demotivated and withdraw care from the patient, asking the hospital to care for them

A member of staff that the others do not like causes problems with motivation for all of the staff.

**Linked Item**

Relationships\\Motivation towards common goal or value (Two way relationship) Relatedness

**Name:** Relationship between relatedness to collaborative behaviour

Relatedness to collaborative behaviours

Several respondents said trust is essential for collaboration.

The CMHT nurses report that because they have a relationship with a client, the client will listen to them and they can plan discharge.

The school counsellor has a relationship with a kid (relatedness) so if the kid is causing problems in class, the teacher will call them (asking for help, respecting the contribution of the other- collaborative behaviours).

The nurse says she gives moral support then secretly tells the patient to see the Bomoh. She is doing this because she cares, but caring together with the hierarchy in this case is causing non-collaborative behaviour - secrecy.

The job support has a relationship with the patient and employer and can make both of them feel supported, which means that they can collaborate.

Collaborative behaviours to relatedness

People get to know and care about each other through collaborating.

Relatedness (eg using the name of the doctor) was frequently associated with describing collaborative behaviour - eg listening.

Carer felt that the doctor didn't care, because he didn't listen. This made the situation worse.

Student counsellors can't care for student (relatedness) because no-one told them they were ill (collaborative behaviour). Lecturers don't know their role (Competency).

The ward staff feel that the orang atas (higher people) do not understand or appreciate what they do. They don't show appreciation (collaborative behaviour), which makes them feel unsupported (relatedness).

**Linked Item**

Relationships\\Relatedness (Two way relationship) Collaborative behaviours

## Additional References

Aein F, Alhani F, Mohammadi E, et al. (2011) Struggling to create new boundaries: A grounded theory study of collaboration between nurses and parents in the care process in Iran. *Journal of Advanced Nursing* 67(4): 841–853. DOI: 10.1111/j.1365-2648.2010.05515.x.

Appleton J V., Terlektsi E and Coombes L (2013) The use of sociograms to explore collaboration in child protection conferences. *Children and Youth Services Review* 35(12). Elsevier Ltd: 2140–2146. DOI: 10.1016/j.childyouth.2013.09.004.

Arnaboldi M and Spiller N (2011) Actor-network theory and stakeholder collaboration: The case of Cultural Districts. *Tourism Management* 32(3). Elsevier Ltd: 641–654. DOI: 10.1016/j.tourman.2010.05.016.

Arsenyan J, Büyüközkan G and Feyzioğlu O (2011) Modelling Collaboration Formation with a Game Theory Approach. *World Congress on Engineering 2011* 1(4): 2073–2085. DOI: 10.1016/j.eswa.2014.10.010.

Beecher B (2009) Mental Health Practitioners’ Views of the Families of Individuals with Schizophrenia and Barriers to Collaboration: A Mixed Methods Study. *Journal of Family Social Work* 12(3): 264–282. DOI: 10.1080/10522150802654260.

Bennett LM and Gadlin H (2012) Collaboration and Team Science: From Theory to Practice. *Journal of Investigative Medicine* 60(5): 768–775. DOI: 10.231/JIM.0b013e318250871d.

Bosque E (2011) A Model of Collaboration and Efficiency Between Neonatal Nurse Practitioner and Neonatologist. *Advances in Neonatal Care* 11(2): 108–113. DOI: 10.1097/ANC.0b013e318213263d.

Daoudi J and Bourgault M (2012) Discontinuity and Collaboration: Theory and Evidence From Technological Projects. *International Journal of Innovation Management* 16(6): 1240012. DOI: 10.1142/S1363919612400129.

Downie J, Orb A, Wynaden D, et al. (2001) A Practice-Research Model for Collaborative Partnership. *Collegian*. DOI: 10.1016/S1322-7696(08)60031-1.

Duffy W (2014) Collaboration (in) Theory: Reworking the Social Turn’s Conversational Imperative. *College English* 76(5): 416–435.

Gajda R (2004) Utilizing collaboration theory to evalute strategic alliances. *American Journal of Evaluation* 25(1): 65–77. DOI: 10.1016/j.ameval.2003.11.002.

Glaser BG and Strauss AL (1967) *The Discovery of Grounded Theory: strategies for qualitative research.* Aldine De Gruyter (ed.). New York.

Guo YH and Feng Y (2010) Research on collaboration theory for design chain. *Proceedings - 2010 International Conference of Information Science and Management Engineering, ISME 2010* 2: 306–309. DOI: 10.1109/ISME.2010.149.

Harley J and Blismas N (2010) An Anatomy of Collaboration Within the Online Environment. In: Anandarajan M and Anandarajan A (eds) *E-Research Collaboration: Theory, Techniques and Challenges*, pp. 15–34. DOI: 10.1007/978-3-642-12257-6.

Huxham C and Vangen S (2004) Doing things collaboratively: Realizing the advantage or succumbing to inertia? *Organizational Dynamics* 33(2): 190–201. DOI: 10.1016/j.orgdyn.2004.01.006.

Jamal T and Stronza A (2009) Collaboration theory and tourism practice in protected areas: stakeholders, structuring and sustainability. *Journal of Sustainable Tourism* 17(2): 169–189. DOI: 10.1080/09669580802495741.

Kaas MJ, Lee S and Peitzman C (2003) Barriers to collaboration between mental health professionals and families in the care of persons with serious mental illness. *Issues in Mental Health Nursing* 24(8): 741–756. DOI: 10.1080/mhn.24.8.741.756.

Mazzola G and Cherlin PB (2009) The Art of Collaboration. In: *Flow, Gesture and Spaces in Free Jazz. Towards a Theory of Collaboration*. Verlag Berlin Heidelberg: Springer, pp. 28–37.

Muijs D, Ainscow M, Chapman C, et al. (2011) *Collaboration and networking in education*. *Collaboration and Networking in Education*. DOI: 10.1007/978-94-007-0283-7.

O’Brien BC, Harris IB, Beckman TJ, et al. (2014) Standards for reporting qualitative research: A synthesis of recommendations. *Academic Medicine* 89(9): 1245–1251. DOI: 10.1097/ACM.0000000000000388.

Price-Robertson R (2012) Interagency collaboration: good in theory, but. *DVRCV Quarterly* (Edition 3 Spring/Summer 2012): 26–29. Available at: http://search.informit.com.au/documentSummary;dn=977997004168492;res=IELFSC.

Ryan RM and Deci EL (2000) Self-determination theory and the facilitation of intrinsic motivation, social development, and well-being. *The American psychologist* 55(1): 68–78. Available at: http://www.ncbi.nlm.nih.gov/pubmed/11392867.

Sawyer RK (2006) Group creativity: musical performance and collaboration. *Psychology of Music* 34(2): 148–165. DOI: 10.1177/0305735606061850.

Sørensen D, Frederiksen K, Groefte T, et al. (2013) Nurse-patient collaboration: A grounded theory study of patients with chronic obstructive pulmonary disease on non-invasive ventilation. *International Journal of Nursing Studies* 50(1). Elsevier Ltd: 26–33. DOI: 10.1016/j.ijnurstu.2012.08.013.

Spinuzzi C (2014) How Nonemployer Firms Stage-Manage Ad-Hoc Collaboration: An Activity Theory Analysis. *Published in Technical Communication Quarterly* 23(232): 88–114. DOI: 10.1080/10572252.2013.797334.

Vangen S (2016) Developing Practice-Oriented Theory on Collaboration: A Paradox Lens. *Public Administration Review* xx: 1–10. DOI: 10.1111/puar.12683.
